# Identification of a novel transthyretin mutation D39Y in a cardiac amyloidosis patient and its biochemical characterizations

**DOI:** 10.3389/fcvm.2023.1091183

**Published:** 2023-01-26

**Authors:** Qunchao Ma, Mengdie Wang, Yanan Huang, Ying Nie, Xin Zhang, Dan Dan Yang, Zhuo Wang, Siyin Ding, Ningjing Qian, Yu Liu, Xiaohong Pan

**Affiliations:** ^1^Department of Cardiology, The Second Affiliated Hospital, Zhejiang University School of Medicine, Hangzhou, Zhejiang, China; ^2^Chinese Academy of Sciences (CAS) Key Laboratory of Separation Science for Analytical Chemistry, Dalian Institute of Chemical Physics, Chinese Academy of Sciences, Dalian, China; ^3^Department of Chemistry, University of Chinese Academy of Sciences, Beijing, China; ^4^Instrumentation and Service Center for Physical Sciences, Westlake University, Hangzhou, Zhejiang, China; ^5^School of Science, School of Life Sciences, Westlake University, Hangzhou, China; ^6^Westlake Laboratory of Life Sciences and Biomedicine, Institute of Natural Sciences, Westlake Institute for Advanced Study, Hangzhou, China

**Keywords:** cardiac amyloidosis, novel mutation, stability, transthyretin, tetramer concentration

## Abstract

Hereditary transthyretin cardiac amyloidosis (hATTR-CA) is a rare autosomal dominantly inherited disease caused by mutations in the transthyretin (TTR) gene. TTR mutations often cause the instability of transthyretin, production of misfolded proteins, and ultimately excessive deposition of insoluble amyloid fibrils in the myocardium, thereby leading to cardiac dysfunction. Herein, we report a novel transthyretin D39Y mutation in a Chinese family. We characterized the kinetic and thermodynamic stabilities of D39Y mutant TTR, revealing that TTR D39Y mutant was less stable than WT TTR and more stable than amyloidogenic mutation TTR L55P. Meanwhile, the only FDA approved drug Tafamidis showed satisfactory inhibitory effect toward ATTR amyloid formation and strong binding affinity in test tube revealed by isothermal titration calorimetry. Finally, we measured the well-folded tetrameric TTR concentration in patient’s and his descents’ blood serum using a previously reported UPLC-based assay. Notably, the tetramer concentrations gradually increased from symptomatic D39Y gene carrier father, to asymptomatic D39Y gene carrier daughter, and further to wild type daughter, suggesting the decrease in functional tetrameric TTR concentration may serve as an indicator for disease age of onset in D39Y gene carriers. The study described a Chinese family with hATTR-CA due to the TTR variant D39Y with its destabilizing effect in both kinetic and thermodynamic stabilities.

## Introduction

Transthyretin cardiac amyloidosis (ATTR-CA) is an infiltrative disease characterized by abnormal deposition of amyloid fibrils in the myocardium (1). ATTR-CA commonly presents with signs and symptoms of heart failure, and is often accompanied by various extracardiac manifestations. The disease can be divided into two categories, wild-type ATTR cardiac amyloidosis (wtATTR-CA) and hereditary transthyretin cardiac amyloidosis (hATTR-CA), according to the presence or absence of a transthyretin (TTR) mutation. Mutations in TTR are the common cause of hATTR-CA. The majority of TTR mutations are caused by single nucleotide substitutions (2). In hATTR-CA patients, the phenotypic presentation, severity and age of onset are variable depending on the specific type of TTR mutation (3). However, ATTR-CA remains a progressive disease with dismal prognosis and poor quality of life. The median survival after diagnosis in patients with Val122Ile hATTR-CA is only 31 months (3). ATTR-CA is often accompanied with racial and geographic differences, yet the clinical and genetic features of this disease are not well elucidated.

Transthyretin consists of 127 amino acids, with a β-sheet-rich structure (3). TTR is synthesized mostly by the liver to transport thyroxine (T4) and holo-retinol binding protein (RBP) in the blood and cerebrospinal fluid (4). Due to hydrophobic and hydrogen bonding, monomeric TTR assembles into tetramer through a dimer intermediate (5). In the case of denaturing microenvironment or genetic mutations, the natural TTR loses its physiological function and conformation to form fibril aggregates that eventually deposit in different organs or tissues (6, 7). Misfolding and aggregation of TTR attract increasing attention, given its amyloidogenicity. This aggregation process occurs in two steps: folded tetrameric TTR dissociates to dimers at the weaker dimer-dimer interface and dimers further dissociate into aggregation-prone monomers; metastable monomers undergo denaturation and eventually assemble into amyloids (8). The tetramers dissociation is the biochemical rate-limiting step of the entire process because the unfolding of monomers is 5–6th orders of magnitude faster than the tetramer dissociation (9). Normally, TTR can induce amyloidosis by decreasing its thermodynamic and/or kinetic stability (10, 11). Thus, studying the biochemical properties of TTR variants is important for the precise diagnosis and choice of therapies for patients with ATTR amyloidosis, especially given their differential clinical symptoms, onset age, and responses to drug treatments.

In this study, we reported a new TTR mutation D39Y occurring in China (c.175G>T p.D39Y, numbered without the first 20 amino acids that serve as signaling peptide and are cleaved during TTR secretion). This patient was a 75-years-old male, presenting dyspnea on exertion and lower extremity edema. Through biochemical and biophysical characterizations, we observed that D39Y displayed compromised thermodynamic stability (Cm = 2.8 M) compared to wild-type TTR (WT-TTR, Cm = 3.4 M). The kinetic stability of D39Y TTR reflected by the tetramer dissociation rate (kdiss = 0.07 h-1, t1/2 = 9.9 h) significantly decreased under physiological conditions. Meanwhile, dissociation of heterotetramers consisting of wild-type and D39Y subunits was not affected by increasing the D39Y mutant subunit ratio, potentially explaining the late disease onset of this mutation. The binding affinity of small molecule drug Tafamidis with TTR-D39Y mutant measured by isothermal titration calorimetry (ITC) was comparable to WT-TTR, resulting in satisfactory inhibition of amyloid formation. Finally, we measured the tetrameric TTR concentration in three blood serum samples across a two-generation pedigree. Notably, the tetramer concentrations gradually increased from symptomatic D39Y gene carrier father, to asymptomatic D39Y gene carrier daughter, and further to wild type daughter, suggesting decrease in functional tetrameric TTR concentration may serve as an indicator for disease age of onset in D39Y gene carriers.

## Materials and methods

### Human samples

A 75-years-old male patient presented relapsed heart failure symptoms and was diagnosed with ATTR-CA at the Department of Cardiology, Second Affiliated Hospital of Zhejiang University College of Medicine (SAHZU). Clinical data were obtained from the medical history system at SAHZU from September 2019 to April 2022. We performed genetic sequencing to differentiate hATTR-CA from wtATTR-CA for this patient. When hATTR-CA is confirmed, we recommend genetic screening for the patient’s 46-year-old daughter and 43-year-old daughter because this disease is inherited. Written Informed consent was taken from the patient and relatives before acquiring blood samples and clinical data. In parallel, blood samples and data analysis were conducted at the SAHZU, and biochemical characterization analysis was performed at Chinese Academy of Sciences (CAS) Key Laboratory of Separation Science for Analytical Chemistry. The participants agreed to the publication of clinical data and complementary examinations after the identifiable personal information was removed. This investigation was performed according to the Declaration of Helsinki and with approval by the Institutional Ethics Committee of SAHZU (Ethical Batch Number: 20220273). This was a prospective study involving retrospective clinical data.

### Plasmid construction and protein purification

Wild-type transthyretin and variants were recombinantly prepared following previous literature. Genes of *E. coli* transthyretin were codon optimized, synthesized by GenScript, and sub-cloned into pET-29b (+) vectors. WT, L55P, and D39Y plasmids were transformed into BL21 DE3 *E. coli* cells. Cells were grown to OD600 at 0.6–0.8 before being induced by IPTG (0.5 mM) at 37°C for 4 h. Cultured cells were harvested and resuspended in resuspension buffer (50 mM Tris, 150 mM NaCl, pH = 7.5; 15 mL buffer/L of culture). Cells expressing recombinant proteins were thawed and lysed by sonication at 4°C. Lysed cells were centrifuged for 30 min at 16,000 rpm. Ammonium sulfate (final concentration of 242 g/L) was slowly added to the supernatant with rigorous stirring at 4°C for 20 min. The solution was centrifuged at 12,000 rpm for 15 min at 4°C. The supernatant was supplemented with additional ammonium sulfate to a final concentration of 607 g/L with rigorous stirring at 4°C for 20 min. The solution was centrifuged at 12,000 rpm for 15 min at 4°C. The pellet was resuspended in 20 mL of anion exchange buffer A (25 mM Tris, 1 mM EDTA, pH = 8.0) and dialyzed against 4 L of buffer A overnight at 4°C. After dialysis, the sample was filtered and applied to a 50 mL Source 15Q anion exchange column (GE Healthcare) equilibrated with buffer A. TTR was eluted using a linear gradient of NaCl (160 mL; 50–350 mM) followed by a NaCl wash (50 mL; 350 mM). Eluted TTR was purified using a 120 mL Superdex 200 gel filtration column (GE Healthcare) in SEC buffer (10 mM sodium phosphate, 100 mM KCl, 1 mM EDTA, pH = 7.4). The protein containing fractions were identified by SDS-PAGE gel analysis, pooled, and concentrated. No significant impurities were identified and purity was estimated to be 98% based on SDS-PAGE electrophoresis analysis.

### Thermodynamic stability of TTR measured by urea-mediated tryptophan fluorescence

To quantify the thermodynamic stability of protein TTR, we denatured D39Y-TTR (3.6 μM) by urea of concentration from 0.5 to 9.0 M in phosphate buffer (10 mM sodium phosphate, 100 mM KCl, 1 mM EDTA, pH = 7.4). After 96 h, the fluorescence of tryptophan was measured from 310 to 410 nm with excitation at 295 nm. The ratio at 355 nm and 335 nm was recorded as a signal of the unfolding of TTR.

### Urea-mediated TTR dissociation measured by resveratrol binding

Resveratrol increases in its fluorescence quantum yield and displays a blue shift when it selectively binds to the TTR tetramer. We quantified the dissociation of TTR by measuring the concentration of tetrameric TTR to text the equilibrium between TTR tetramers and monomers. 500 μL D39Y TTR (3.6 μM) was incubated with urea (5 M) in phosphate buffer (10 mM sodium phosphate, 100 mM KCl, 1 mM EDTA, pH = 7.4) for 96 h. In order not to disturb the equilibrium of tetramers and monomers, TTR samples were treated with resveratrol (18 μM) just before the fluorescence measurement. Excited at 320 nm, the resveratrol fluorescence spectrum was recorded from 310 to 410 nm. Fluorescence at 394 nm was reported on the concentration of tetrameric D39Y TTR.

### Kinetics of monomer unfolding and tetramer dissociation measured by urea denaturation

Kinetic stability was determined by the rate-limiting dissociation of the tetramer, because monomer unfolding was much faster than tetramer dissociation. To verify this, we incubated TTR (3.6 μM) in urea at the concentration from 3 to 9 M in phosphate buffer (10 mM sodium phosphate, 100 mM KCl, 1 mM EDTA, pH = 7.4, 25°C). Tryptophan fluorescence (I355/335) was recorded at period of time. The kinetics data fit well to a single exponential function: I355/335 = I355/335N + A (1–e-kdiss t); where I355/335N is the native protein fluorescence intensity ratio (355/335 nm), A is the amplitude difference, kdiss is the tetramer dissociation rate constant and t is time in h. The lnkdiss versus urea concentration plot is linear, allowing extrapolation to 0 M urea.

### Fibril formation assay

Tafamidis of various concentrations was preincubated with TTR (7.2 μM) in phosphate buffer (10 mM sodium phosphate, 100 mM KCl, 1 mM EDTA, pH = 7.4) at 37°C for 0.5 h. Acidic aggregation buffer (200 mM NaOAc, 100 mM KCl, acidified by AcOH to pH = 4.40) was added to the samples to get a final concentration of TTR of 7.2 μM, and after incubating at 37°C for 72 h, the extent of fibril formation was measured by OD at 330 nm.

### Isothermal titration calorimetry (ITC)

Following the protocol reported before, dissociation constants for Tafamidis binding to wild type, L55P and D39Y TTR were determined using a MicroCal PEAQ-ITC (Malvern MAN0572-01-EN-00). A solution of Tafamidis (300 μM in 10 mM sodium phosphate, 100 mM KCl, 1 mM EDTA, 5% DMSO, 5% EtOH, pH = 7.4) was prepared and titrated into an ITC cell containing TTR (25 μM in 10 mM sodium phosphate, 100 mM KCl, 1 mM EDTA, 5% DMSO, 5% EtOH, pH = 7.4). The initial injection of 1 μL was followed by 35 injections of 1 μL each (25°C). Integration of the thermogram after subtraction of blanks yielded a binding isotherm that fit best to a model of one binding site. The data were fit by a non-linear least squares approach with one adjustable parameter, namely, Kd, using the ITC data analysis module in Microcal PEAQ-ITC analysis software.

### Analysis of patients serum by ultra performance liquid separation (UPLC)

This protocol was adapted from previous work by the Kelly group. Briefly, UPLC (FLR) Waters Acquity H-Class uplc plus pro and Protein-pakTM Hi RCSQ (5 μm, 4.6 × 100 mm) were from Waters. The A2 molecule was synthesized in the laboratory. The patients’ sera were incubated with A2 for covalent labeling and subjected to UPLC separation and detection of tetramer TTR. Each sample was injected onto a Waters Acquity H-Class UPLC plus pro instrument fitted with a Protein-pakTM Hi RCSQ (5 μm, 4.6 × 100 mm) from Waters. TTR was eluted from the column using the following gradient (Buffer A: 25 mM Tris–HCl, pH = 9.0, 1 mM EDTA; Buffer B: 1 M NaCl, 1 mM EDTA, 25 mM Tris–HCl, pH = 9.0).

## Results

### Genetic mutation identification and patient’s clinical presentations

**FIGURE 1:**
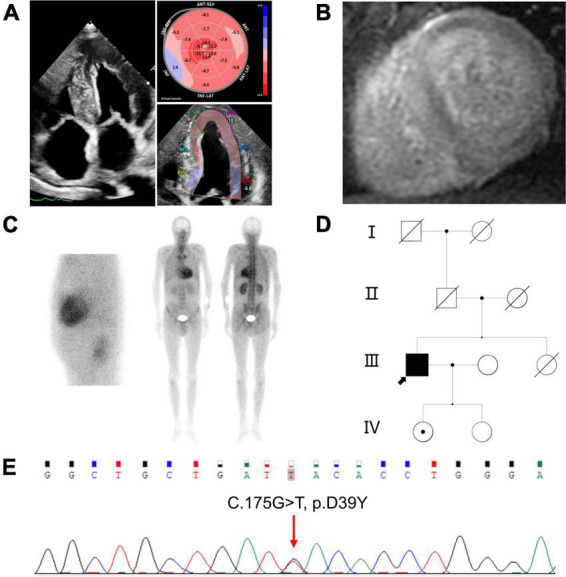
Clinical features of the proband **(A)** right, 4-chambers view of two-dimensional echocardiography; left, speckle tracking echocardiography; **(B)** short axis view of cardiac magnetic resonance imaging showed diffuse subendocardial late gadolinium enhancement in ventricular walls; **(C)** Tc-99m-PYP scintigraphy revealed grade 3 myocardial uptake, and HCL ratio was 2.38; left, planar myocardial imaging; right, planar whole-body imaging; **(D)** pedigree of the family. The black arrow represents the proband, dot in the center of the circle represent female carrier: **(E)** Sequence analysis of exon 2 of the TTR gene. The proband showed a homozygous missense variant with a G>T change at nucleotide 175. PYP: Pyrophosphate. HCL: Heart/contralateral lung ratio. TTR, Transthyretin.

The proband presented worsening dyspnea on exertion and lower extremity edema over 1 year. The proband’s ECG revealed an atrial flutter with low voltage in the limb leads, with a QS pattern in lead II, III and aVF. Laboratory investigations showed mild elevated BNP (135.7 pg/ml) and troponin T (0.027 ng/ml). Serum and urine protein electrophoresis with immunofixation and sFLC assay were negative. Echocardiography showed bi-atrial enlargement, symmetrical left ventricular hypertrophy (septal 17.4 mm and posterior wall 16.6 mm), and mild impaired left ventricular systolic function (LVEF 49.2%) (Figure 1A, right side). Speckle tracking echocardiography revealed severely depressed global longitudinal strain with apical sparing (GLS-8.4%) (Figure 1A, left side) which was a hallmark of cardiac amyloidosis. Cardiac enhanced magnetic resonance showed diffuse subendocardial late gadolinium enhancement (Figure 1B). Tc-99m-PYP nuclear scintigraphy showed grade 3 myocardial uptake with increased heart/contralateral lung ratio (HCL 2.38) (Figure 1C). Furthermore, the proband and his two asymptomatic daughters were sequenced and analyzed (Figure 1D). The proband showed a novel homozygous missense variant at base position 175 in exon 2 of the TTR gene (c.175G>T), resulting in an aspartic acid change to tyrosine (p.D39Y, numbered without the first 20 amino acids that serve as signaling peptide and are cleaved during TTR secretion) (Figure 1E). The proband’s eldest daughter was identified as carrying this heterozygous variant of TTR gene c.175G>T. *In silico* analyses predicted this c.175G>T variant could be pathogenic (SIFT: affect protein function; Polyphen-2: benign; Mutation Taster: polymorphism; M-CAP: damaging).

### Thermodynamic stability of the TTR variant D39Y

**FIGURE 2:**
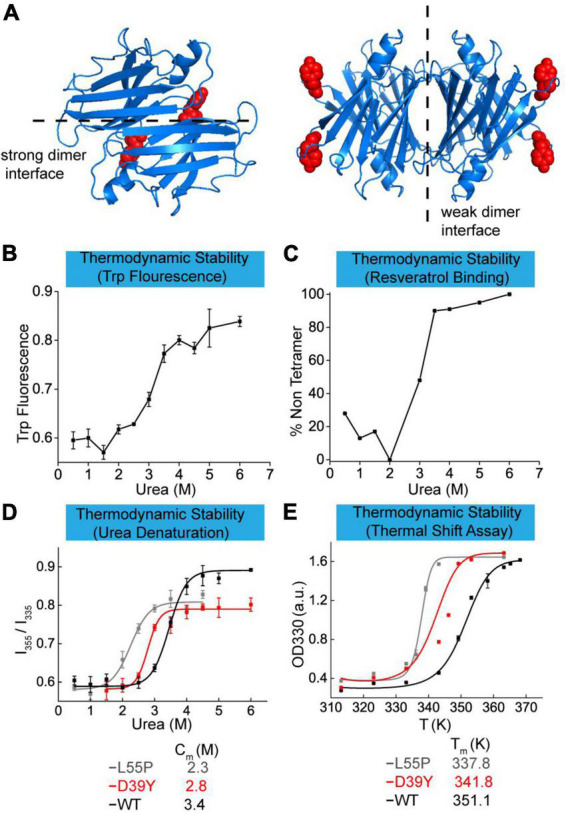
Thermodynamic stability of TTR-D39Y. **(A)** X-ray crystallographic structure of D39Y-TTR modified from WT-TTR (PDB code: 1BMZ). Y-39 was highlighted in red. **(B)** Urea-mediated unfolding of TTR-D39Y measured by tryptophan fluorescence changes (ratio at 355 nm and 335 nm). **(C)** Urea-mediated tetramer dissociation of TTR-D39Y measured by resveratrol binding fluorescence (394 nm). **(D)** Denaturation curve measured by tryptophan fluorescence. **(E)** Thermodynamic stability measured by thermal shift assay.

Point mutants on TTR protein usually affect its thermodynamic and kinetic stability leading to ATTR amyloidosis diseases. In this work, we elucidated the effect of D39Y mutation (Figure 2A) on the thermodynamic stability in three ways and compared it with the parameters of commonly studied mutation L55P and wild type TTR. First, we measured the thermodynamic stability by urea-mediated denaturation (Figure 2B), followed by resveratrol binding measurement (Figure 2C). Normally, the thermodynamic stability was quantified by urea denaturation midpoints (Cm), revealing that D39Y (Cm = 2.8 M) exhibited higher stability than L55P-TTR (Cm = 2.3 M), but lower than that of WT-TTR (Cm = 3.4 M) (Figure 2D). To further validate this result, we performed a thermal shift assay that can quantify the thermodynamic stability of TTR *via* melting temperature (Tm). TTR (7.2 μM) was heated in acid aggregation buffer (NaOAc 200 mM, KCl 100 mM, acidified by AcOH to pH = 4.4), respectively from 35 to 96°C for 5 min and measured the optical density at 330 nm. The thermal shift assay concluded the same that D39Y (Tm = 341.8 K) is more stable than L55P (Tm = 337.8 K), but less stable than WT (Tm = 351.1 K) and (Figure 2E). Overall, D39Y-TTR is thermodynamically destabilized compared to WT-TTR.

### Kinetic stability of D39Y-TTR mutant protein

**FIGURE 3:**
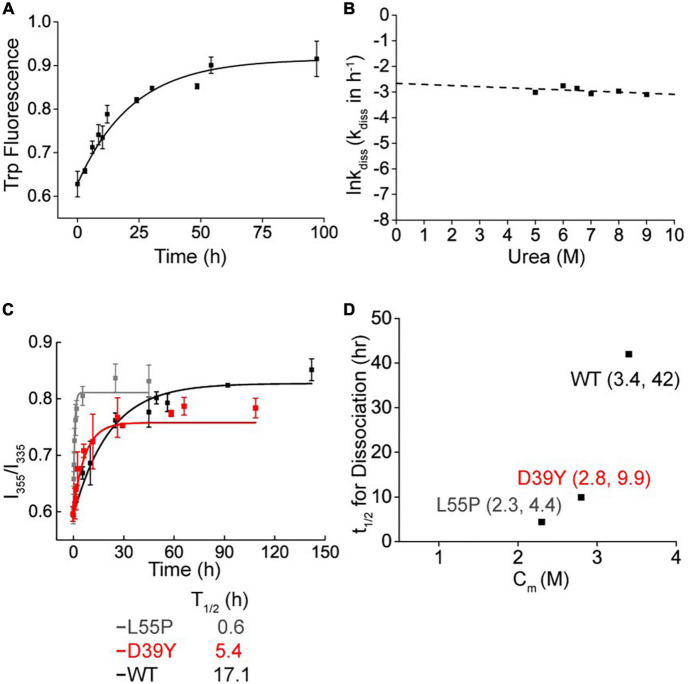
Kinetic stability of D39Y TTR. **(A)** Tetramer dissociation measured by tryptophan fluorescence in 9 M urea. **(B)** The logarithm of the rate of tetramer dissociation, lnkdiss (kdiss in h-1) plotted as a function of urea concentration. The lnkdiss vs. urea concentration plot is linear, allowing extrapolation to 0 M urea. **(C)** Kinetic stability measured by tryptophan fluorescence in 6 M urea. L55P, gray curve; D39Y, red curve; WT, black curve. **(D)** Relationship between thermodynamic (*x* Axis; Cm of denaturation curve) and kinetic (*y* Axis; t1/2 of tetramer dissociation) stability.

Some TTR mutations destabilize TTR by decreasing the tetramer dissociation kinetic barrier. To verify whether the variant D39Y reduced the kinetic stability of TTR, we quantified it using tetramers dissociation half time (t1/2) measured by urea mediated denaturation (Figures 3A, B). Kinetically, in 6 M urea, D39Y (t1/2 = 5.4 h) was less stable than WT (t1/2 = 17.1 h), but more stable compared with L55P (t1/2 = 0.6 h) (Figure 3C). Under physiological conditions (0 M urea), the conclusion still held true. Extrapolated from the urea mediated tetramer dissociation curve (Figure 3B), D39Y (t1/2 = 9.9 h, 0 M urea) was less stable compared with WT (t1/2 = 42 h), but more stable than L55P (t1/2 = 4.4 h) on kinetic stability under 0 M physiological conditions (Figure 3D).

### D39Y: WT TTR heterozygous tetramers is kinetically stable and resistant to proteolysis

**FIGURE 4:**
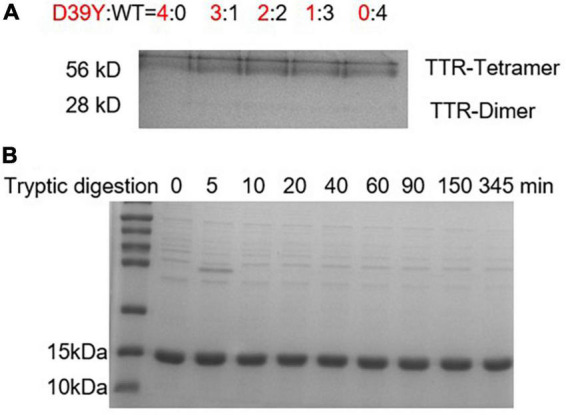
Heterozygous tetramers of D39Y: WT TTR is stable and resistant to proteolysis. **(A)** SDS-PAGE electrophoresis analysis of D39Y: WT TTR heterozygous tetramers after crosslinking revealed no increasing dimer bands upon increasing D39Y mutant protein ratio. It suggests heterozygous tetramers of D39Y: WT TTR is kinetically stable. Heterozygous tetramers were prepared at indicated molar ratios of D39Y mutant (red) to WT (black), from 4:0 (D39Y alone) to 0:4 (WT alone), as shown above the gel. **(B)** SDS-PAGE electrophoresis analysis of D39Y showed proteolytic resistance toward trypsin digestion, suggesting D39Y mutation does not generate proteolytic fragments that lead to amyloid formation.

Transthyretin is usually heterotetramers in patients’ serum due to the subunit exchange effect. To compare the kinetic stability of heterotetramers of variant D39Y and WT, we mixed up D39Y and WT TTR and incubated them in phosphate buffer (10 mM sodium phosphate, 100 mM KCl, 1 mM EDTA, pH = 7.4) at D39Y:WT ratios from 4:0 to 0:4 at 4°C for 48 h for efficient subunit exchange. Then, the subunit exchanged samples were subjected to cross-linking reaction. Through the cross-linking reaction, glutaraldehyde crosslinked two TTR monomers into a dimer, the dimer into a tetramer, and the dimer into a tetramer, as well as octamers and multimers of larger molecular weights. Therefore, we observed these products of higher molecular weights as corresponding bands on this SDS-PAGE gel. Due to the limited resolution of the SDS-PAGE gel and the randomness of crosslinking reaction between TTR multimers, these higher molecular bands representing TTR crosslinked multimers exhibited as a smear bands cluster at the top of the gel. Fortunately, this experiment does not rely on the quantification of these smear bands of higher molecular weights. We monitored the dimeric bands that were well defined and the molecular weight of which was clearly discernible by the gel ladder. SDS-PAGE gel analysis of TTR proteins after cross-linking showed that homotetramers and heterotetramers had the same kinetic stability as the dissociated dimer bands did not change with the increasing ratio of D39Y and WT (Figure 4A). This result may explain the late disease age-of-onset for the D39Y patient reported herein.

On the other hand, besides the deposition of full-length amyloid TTR, fragment 49–127 to trigger TTR aggregation is also a pathway to form amyloid fibrils. To assess whether D39Y is capable of forming digested fragments, D39Y (3.6 μM) was incubated with trypsin (5 μg/mL) at 37°C for 0–345 min. The products were analyzed by SDS-PAGE gel, revealing no digested bands at 9 kDa. This result demonstrates that D39Y is resistant to proteolysis and does not generate amyloid-prone proteolytic fragments (Figure 4B). Overall, these lines of evidence may also explain D39Y late disease age-of-onset.

### D39Y responses well to tafamidis stabilization

**FIGURE 5:**
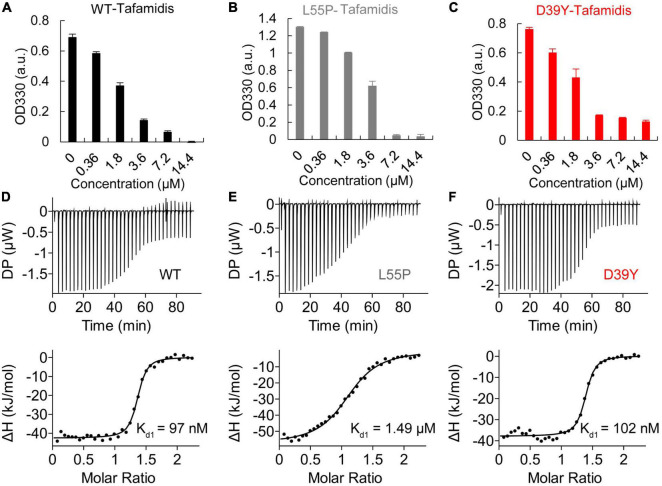
Inhibitory effect of Tafamidis on TTR amyloid formation. **(A)** WT **(B)** L55P **(C)** D39Y (3.6 μM) were incubated with increasing concentration of Tafamidis in acidic aggregation buffer (200 mM NaOAc, 100 mM KCl, acidified by AcOH to pH = 4.4) for 72 h. OD330 turbidity assay was used to monitor the formation of TTR amyloid. Isothermal titration calorimetry (ITC) of Tafamidis with wild type **(D)**, L55P **(E)** and D39Y **(F)** TTR revealed the dissociation constant of Tafamidis with TTR. TTR concentrations were 300 μM.

We next evaluated whether D39Y TTR exhibited a differential response to Tafamidis stabilization compared to WT and L55P-TTR. Using OD300 turbidity assay, we assessed the inhibitory effect of Tafamidis on WT-TTR and mutant TTR amyloid formation under acidic aggregation conditions (200 mM NaOAc, 100 mM KCl, acidified by AcOH to pH = 4.4). D39Y showed a similar response to Tafamidis upon one site occupation at 1:1 stoichiometry (Figures 5A–C). However, upon the increasing concentration of Tafamidis, we did not observe a dose-dependent inhibitory effect after 3.6 μM, indicating its negative cooperativity present in mutant TTR binding to Tafamidis. This result was supported by the ITC experiment as two binding site model was not feasible in D39Y mutant (Figures 5D–F). However, D39Y binds to Tafamidis as tightly as the WT TTR, indicating its potential satisfactory response to Tafamidis treatment.

### Functional tetrameric TTR concentration in patient’s serum indicates the disease age-of-onset

**FIGURE 6:**
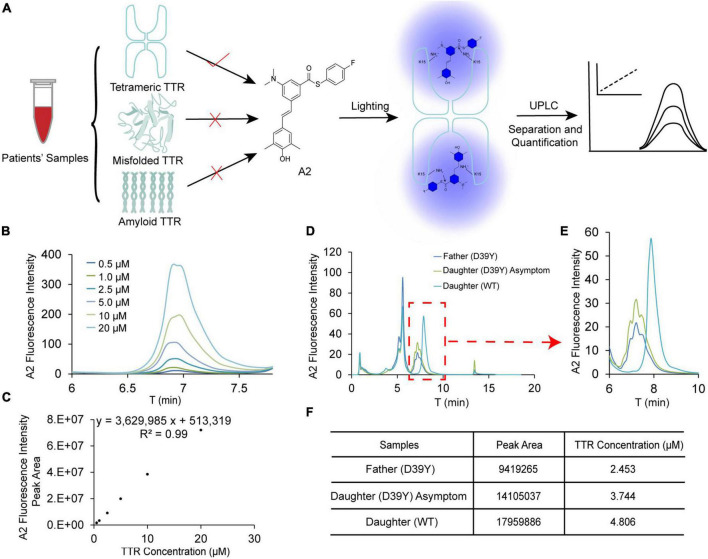
Quantification of tetramer TTR concentration in patient’s blood serum. **(A)** The scheme for quantification of TTR tetramer concentration in patient’s serum using by UPLC. A2 selectively reacts with TTR tetramer and reports on the concentration by its fluorescence. **(B,C)** Linear correlation between TTR concentration and fluorescence intensity of A2. **(D)** The raw chromatographic data for the symptomatic father (D39Y), asymptomatic daughter (D39Y) and healthy daughter (WT) on UPLC detected by A2 fluorescence. **(E,F)** Quantification of the peak area and TTR tetramer concentration of three samples.

We next explored the tetramer concentration of TTR in patient’ serum using UPLC method reported by the Kelly group (11) (Figure 6A). Stilbene A2 probe selectively reacts with the Lysine-15 at the weak dimer-dimer interface of TTR tetramer and fluoresces at 430 nm. Due to the stable covalent bonds between A2 and TTR tetramers, we can quantify the folded tetrameric TTR by UPLC (Figure 6A). Based on the above working principle of this assay, we established the linear correlation between the fluorescence intensity of the peak and TTR tetramer concentration spiked in serum for afterward quantification (Figures 6B, C). The results showed that A2 fluorescence intensity increased with the concentration of TTR, giving a wide linear range from 0.5 to 20 μM (Figure 6C).

It has been reported that TTR may partition into folded tetrameric, misfolded oligomeric and amyloid fibril conformations. The decrease in tetramer TTR in blood serum may manifest the disease onset of TTR amyloidosis. Toward this end, we assessed the concentration of TTR tetramer in D39Y symptomatic patient (father), asymptomatic D39Y gene carrier (daughter 1), and wild type non-D39Y gene carrier (daughter 2). Next, we quantified the TTR tetramer concentration in the sera of three subjects of the patient’s family (Figures 6D, E). Intriguingly, the fluorescence intensity of the healthy daughter (WT, 4.8 μM) shows much higher than the symptomatic father (D39Y, 2.5 μM). Whereas the asymptomatic daughter’s TTR tetramer concentration stayed in the middle (D39Y, 3.7 μM) (Figure 6F). In addition, the chromatographic peak shape of D39Y (multiplet peaks) and WT (singlet peak) TTR in this assay reflects its genotypes. These results highlighted that TTR tetramer concentration may serve as a disease onset parameter for D39Y mutation carrier and for the drug interference window time.

## Discussion

Cardiac amyloidosis is a devastating and progressive infiltrative cardiomyopathy caused by the extracellular deposition of misfolded transthyretin (12). The clinical presentation and manifestations are non-specific in early stage amyloidosis, leading to massively underdiagnoses. Early diagnosis and treatment were essential for preventing disease progression and adverse cardiovascular events. Witteles et al. (13) had elaborated that under what clinic scenarios should amyloid cardiomyopathy be highly suspected and associated with “Red-flag” signs. In this study, we described the first case of hATTR-CA associated with a D39Y TTR mutation in China. The proband was admitted to our hospital with a series of “Red-flag” features, such as signs and symptoms of heart failure, limb lead low voltage on EKG and increased left ventricular wall thickness on echocardiography. Thus, we initiated screening for amyloidosis in this patient.

The amyloidogenic risk factors for a TTR mutant are usually associated with thermodynamic and kinetic stability (14–17). Thermodynamic stability and kinetic stability measurements of the TTR D39Y mutant showed that it was less stable than WT TTR and more stable than amyloidogenic mutation TTR L55P. These data indicated that instability of variant D39Y TTR is one of the fundamental causes of TTR amyloidosis. TTR are heterotetramers in patients’ serum due to the subunit exchange effect. We further revealed the heterozygous tetramer is stable, implicating D39Y’s late onset amyloid cardiomyopathy. Despite tetramer destabilization being generally considered to be the major step in developing amyloid fibrils, it has recently been shown that TTR proteolysis has been suggested as another mechanism mediating the amyloid formation (18, 19). The TTR fragments, particularly the 49–127°C-terminal peptide contributed to the formation of TTR amyloid fibrils (18, 19). Interestingly, we did not detect the amyloid-prone proteolytic fragments generated by D39Y variant. This might be because some amyloid variants are insensitive to this limited proteolysis *in vitro* (13, 20). In heart tissue with shear force, these variants become more susceptible to proteolytic cleavage and decomposition under shear stress, which seems to explain why our proband had the cardiac-predominant phenotypes (18, 20).

The average concentration of serum TTR ranged from 3 to 6 μmol/L in healthy adult humans (21–23). The levels of serum TTR found in previous studies are reduced in hATTR patients (24, 25). The decline in serum TTR portended worse survival in wild-type ATTR-CA patients, which was considered as an independent predictor of OS in patients with transthyretin amyloidosis (26). A Danish cohort study including 16,967 individuals demonstrated that lower serum TTR concentrations indicated a risk of incident HF (27). Also, patients with higher tetrameric TTR tend to respond better to TTR kinetic stabilizers (28–30). Here, The A2-UPLC quantification method was established to detect the tetrameric plasma TTR under native conditions. Among family members, the proband had the lowest TTR concentration with symptoms of heart failure. His asymptomatic daughter had a mild decrease in TTR concentration compared to his non-carrier daughter. Hence, the variant destabilizes the TTR tetramer structure, contributing to amyloid deposition. However, given the small sample size, we cannot wholly exclude the influence of other factors (e.g., age, gender, and nutritional status), which might interfere with the TTR concentration. Additionally, periodic monitoring to observe dynamic changes in the TTR tetramer concentration should be considered in further studies.

More than 120 pathogenic variants have been identified in the TTR gene (2). The Val30Met is the most frequent pathogenetic variant in the Europe population and Japan (12, 31). This mutation is typically associated with familial amyloid polyneuropathy, whereas approximately 43% of patients have cardiac involvement (31). The val122Ile variant is the most common cause of hATTR-CA (2, 32). However, Damrauer et al. (33) showed that among 92 TTR val122Ile carriers with heart failure, 24 (26%) experienced neuropathy, while only 13 (14%) suffered from carpal tunnel syndrome. This finding suggested the necessity of genetic screening for TTR patients. Currently, only scattered TTR variants were found in China, and most of them were associated with familial amyloid polyneuropathy, lacking comprehensive analysis and diagnosis of cardiac amyloidosis (34–37). In this study, the proband and his asymptomatic daughter carried a novel missense variant in TTR D39Y. The proband presented with hATTR-CA demonstrated a clinical profile dominated by cardiac manifestations, largely without evidence of extracardiac manifestations (e.g., polyneuropathy, carpal tunnel syndrome, autonomic insufficiency, and gastrointestinal dysfunction). The exact age of onset and penetrance of this D39Y mutant is not fully elucidated, which could account for this heterogeneity of phenotype.

Recently, several new drugs targeting TTR stabilization and suppressing TTR production have been developed for ATTR amyloidosis therapy. Tafamidis, an oral drug that acts as a small molecule stabilizer of TTR tetramers has been approved for the treatment of familial amyloid polyneuropathy (FAP) and ATTR-CA (38–40). The Tafamidis in Transthyretin Cardiomyopathy Clinical Trial (ATTR-ACT) enrolled 441 patients with ATTR cardiomyopathy, and 106 (24%) of them were hATTR-CA patients with the most common mutations of Val122Ile, Thr60Ala, and Ile68Leu (40). This trial showed that Tafamidis treatment reduced mortality and cardiovascular-related hospitalizations, improved quality of life and relieved symptoms in patients with ATTR-CA compared to placebo controls. Of note, our study verified Tafamidis had a profound inhibition on WT-TTR and L55P TTR amyloid formation. D39Y showed a similar response upon Tafamidis treatment. Also, the ITC experiments revealed that the binding affinities of Tafamidis with D39Y are similar to WT-TTR and much stronger than L55P. Here, we demonstrated the potential benefits of Tafamidis D39Y hATTR-CA. Despite encouraging preliminary results, further studies including long term follow-up are required.

## Conclusion

In conclusion, this study described a Chinese family with hATTR-CA due to the TTR variant D39Y with its destabilizing effect in both kinetic and thermodynamic stabilities. Also, a decrease in functional tetrameric TTR concentration may serve as a disease age of onset indicator for the D39Y gene carriers. Tafamidis also exhibited the potential benefits to D39Y hATTR-CA.

## Data availability statement

The datasets presented in this study can be found in online repositories. The names of the repository/repositories and accession number(s) can be found below: http://doi.org/10.17632/4cpkx3 c66b.1, CC BY 4.0.

## Ethics statement

The studies involving human participants were reviewed and approved by Institutional Ethics Committee of Second Affiliated Hospital of Zhejiang University School of Medicine. The patients/participants provided their written informed consent to participate in this study. Written informed consent was obtained from the individual(s) for the publication of any potentially identifiable images or data included in this article.

## Author contributions

XP and YL designed the experimental study and revised the manuscript. QM provided the clinical samples and analyzed clinical data. MW and YH conducted experiments and acquired data. YN and XZ acquired the ITC results. QM and MW performed the data analyses and wrote the manuscript and prepared the figures. DY and ZW compiled the imaging data collection and consultation. SD and NQ helped with patient follow-up. All authors read and approved the final manuscript.
